# Identifying in-market application of Pelargonium root extract EPs 7630 for the treatment of COVID-19: analysis of pharmacovigilance data

**DOI:** 10.3389/fphar.2024.1335309

**Published:** 2024-02-23

**Authors:** Fathi Abdul Malek, Petra Funk

**Affiliations:** Research and Development, Dr. Willmar Schwabe GmbH & Co. KG, Karlsruhe, Germany

**Keywords:** Pelargonium sidoides root extract (EPs 7630), off-label use, case reports, COVID-19, spontaneous reporting, safety, effectiveness

## Abstract

**Introduction:** Phytopharmaceutical products are successfully used for acute respiratory infections and may therefore be promising candidates for adjuvant symptomatic treatment of COVID-19. *In vitro* and clinical studies suggest that the proprietary *Pelargonium sidoides DC.* root extract EPs 7630 has antiviral and immunomodulatory properties, and effects on SARS-CoV-2 propagation have been shown *in vitro*. Medicinal products containing the extract have been approved for the symptomatic treatment of acute viral respiratory tract infections.

**Methods:** We present a retrospective review of case reports submitted spontaneously to the pharmacovigilance database of the manufacturer of EPs 7630 and containing information on the off-label use of the extract for the treatment and prophylaxis of COVID-19 and of post-COVID-19 syndrome. Eligible case reports were identified by automated database searches.

**Results:** Forty-four case reports filed between December 2019 and February 2023 were eligible for analysis. More than ¾ described the use of EPs 7630 for treatment of COVID-19 while the remaining reports referred to the treatment of post-COVID-19 syndrome or to COVID-19 prophylaxis. 15/22 cases which reported on treatment duration indicated an intake of EPs 7630 for up to 7 days. Five case reports indicated the use of EPs 7630 as COVID-19 monotherapy while 14 indicated a combination treatment with other drugs. All 28 cases that reported on treatment outcome characterized the patients as improved. Thirty case reports (68%) did not indicate any complications. The most frequent suspected adverse reactions were gastrointestinal complaints and hypersensitivity reactions, both of which may occur as known adverse effects of EPs 7630. No unexpected adverse reactions were observed.

**Conclusion:** Reported cases confirm that there was a certain off-label use of EPs 7630 for COVID-19 in the market. Even though no formal conclusions about the efficacy of EPs 7630 in COVID-19 can be drawn, a beneficial effect would be explainable by the pharmacological profile of the extract. Further assessment of the effects of EPs 7630 in COVID-19-related indications therefore appears to be both justified and promising, particularly as the available case reports did not give rise to any safety concerns also in this patient group.

## 1 Introduction

The occurrence of the severe acute respiratory syndrome coronavirus 2 (SARS-CoV-2)/coronavirus disease 2019 (COVID-19) has motivated an intensive search for existing treatments with proven efficacy in other viral respiratory diseases to identify promising candidates for investigating whether they may have a beneficial effect in patients with COVID-19. This includes the herbal extract EPs 7630 (EPs^®^ 7630 is a proprietary extract and the active ingredient in pharmaceuticals manufactured by Dr. Willmar Schwabe GmbH and Co. KG).

EPs 7630 is a hydroethanolic extract from the roots of *Pelargonium sidoides* DC., drug-extract ratio 1:8–10, extraction solvent ethanol 11% (w/w). The plant material must contain not less than 2% of tannins as defined in the monograph of the European Pharmacopoeia. EPs 7630 can be classified according to the European Pharmacopoeia as an “other extract”. Therefore, it is not adjusted to a specific content of constituents. Oligomeric prodelphinidins are the most significant group of plant ingredients which account for approximately 40% of the dried extract, other groups of constituents are carbohydrates, minerals, peptides, purine derivatives and highly substituted benzopyranones (Schötz et al., 2008).

*Pelargonium sidoides* DC. (Geraniaceae), also known as African geranium or South African geranium, is a plant native to South Africa, which shares the same genus (*Pelargonium*) with the common ornamental geraniums. *Pelargonium sidoides* became known in Europe when it was described by Charles H. Stevens and its root praised as a new remedy for tuberculosis around 120 years ago (Bladt and Wagner, 2007). Today, medicinal products containing *Pelargonium sidoides* extract EPs 7630 have been approved in several countries in Europe, Asia, Australia, and Central as well as South America. Approved therapeutic indications include acute respiratory tract infections such as the common cold (CC), acute bronchitis (AB), acute rhinosinusitis, and acute tonsillopharyngitis in adults, adolescents, and children from the age of 1 year. The marketed medicinal product is available as a solution, as film-coated tablets, and as a syrup for children. The recommended daily doses are 3 × 30 drops of solution or 3 × 1 tablet containing 20 mg of the herbal extract, corresponding to the quantity contained in 30 drops of EPs 7630 solution, for children and adolescents older than 12 years and adult patients, as well as EPs 7630 solution 3 × 10 drops/day for children 1–5 years of age and 3 × 20 drops/day for children 6–12 years of age. 2.5 mL of syrup are equivalent to 10 drops of the solution.

*In vitro* experiments with EPs 7630 and isolated constituents showed pharmacological activities including notable immune-modulatory capabilities and moderate direct antiviral and antibacterial effects: The immune-modulatory capabilities observed involve the activation of the mitogen-activated protein kinase pathway (Witte et al., 2015) and a subsequent regulation of different cytokines (Kolodziej et al., 2003; Witte et al., 2020). The extract was also shown to interfere with the replication of seasonal influenza A virus strains (H1N1, H3N2), respiratory syncytial virus, human coronavirus 229E, parainfluenza 3 virus, and coxsackie virus A9 (Michaelis et al., 2011). Moreover, EPs 7630 reduced rhinovirus infection of human bronchial cells (Roth et al., 2019). This effect was associated with a downregulation of cell-membrane-docking proteins and an upregulation of host defense proteins (Roth et al., 2019), and also an increased expression and nuclear translocation of vitamin D receptor cells (Roth et al., 2021). Moreover, EPs 7630 was shown *in vitro* to improve the recovery of human airway epithelial cells by stimulating tissue repair mechanisms and modifying tight junction protein expression, also in RV16-infected epithelial cells (Fang et al., 2023).

The effects of EPs 7630 on SARS-CoV-2 propagation and the innate immune response in the human lung cell line Calu-3 were also assessed *in vitro* (Papies et al., 2021a; Papies et al., 2021b) and it was shown that, in direct comparison to the other highly pathogenic coronaviruses SARS-CoV and MERS-CoV, SARS-CoV-2 growth was most efficiently inhibited by EPs 7630. In particular, the cellular entry step of SARS-CoV-2 was significantly reduced by EPs 7630 pretreatment (Papies et al., 2021a; b). Assessment of cytokine secretion in EPs 7630-treated and SARS-CoV-2-coinfected Calu-3 cells showed that the pro-inflammatory cytokines IL-1β and IL-6 were elevated, while a range of other COVID-19-associated cytokines (IL-8, IL-13, TNF-α), chemokines (CXCL9, CXCL10), and growth factors (PDGF, VEGF-A, CD40L) were significantly reduced (Papies et al., 2021a; b). *In vitro* inhibitory activity against SARS-CoV-2 was also shown for a commercial form of EPs 7630 and for the biomolecules scopoletin and umckalin contained in the extract (Alossaimi et al., 2022).

*In vivo*, a significant, dose-dependent reduction of cough frequency was observed in animal cough models (Bao et al., 2015). EPs 7630 further exerted a marked secretolytic activity in mice and reduced lesions of lung tissue adjacent to trachea and bronchi in rats with experimentally induced bacterial bronchitis (Bao et al., 2015). In a hamster model, Emanuel et al. (2023) demonstrated that EPs 7630 reduced viral load early in the course of infection and displayed significant immunomodulatory properties positively modulating disease progression. The authors also observed an inhibition of SARS-CoV-2 variants in primary nasal and bronchial human airway epithelial cells, with particularly pronounced antiviral effects against the Omicron BA.2 variant (Emanuel et al., 2023).

Moreover, using classical molecular dynamics simulation, Iacovelli et al. (2022) were able to show that *P. sidoides* extract may interfere with the mechanism of infection of SARS-CoV-2, especially in the early stages.

Clinically, EPs 7630 was shown to modulate the innate immune system through the proliferation of NK cells in an examination of its effect on T-CD8 lymphocytes and natural killer cells (NK) proliferation in peripheral blood mononuclear cells (PBMC) from 12 healthy individuals (Polisello et al., 2022). EPs 7630 also exerted modulatory effects on the production of chemokines regulating the function of neutrophils and monocytes in the nasal mucosa in an observational, prospective, case-control study with patients suffering from acute, post-viral rhinosinusitis (Perić et al., 2020).

The existing double-blind, randomized, controlled trials on efficacy and safety of EPs 7630 in acute, viral respiratory disorders were summarized and evaluated in several reviews and meta-analyses (Matthys et al., 2013; Matthys et al., 2016; Schapowal et al., 2019; Seifert et al., 2019; Seifert et al., 2021; Kardos et al., 2022; Matthys et al., 2023). Compared to placebo, the authors consistently observed significant advantages of EPs 7630 for the efficacy outcomes assessed, i.e., the primary outcomes as assessed by disease-specific symptom scores (Matthys et al., 2016; Schapowal et al., 2019; Kardos et al., 2022) and the secondary outcomes time to substantial improvement or complete remission (Matthys et al., 2016; Schapowal et al., 2019; Seifert et al., 2019), disease-associated quality of life (QoL; Kardos et al., 2022), concomitant use of paracetamol (Matthys et al., 2016; Seifert et al., 2019; Seifert et al., 2021), and days missed at work or school (Schapowal et al., 2019; Seifert et al., 2019; Seifert et al., 2021; Matthys et al., 2023). Moreover, a systematic review and network meta-analysis demonstrated that *P. sidoides* extract is effective in improving the symptoms of the common cold and of acute post-viral rhinosinusitis with moderate certainty of evidence (Hoang et al., 2023). However, as for all herbal medicines analyzed in the latter review, the authors could find only very low certainty of evidence for reduction of cold symptoms compared to placebo.

As shown by a comprehensive review on the available evidence on the safety and tolerability profile of EPs 7630 from clinical trials and non-interventional studies, including data from 10,026 adults and children, the extract appears to be a well-tolerated herbal medicine in the management of respiratory tract infections in adults and children (Matthys et al., 2013). The good safety and tolerability were also demonstrated both by a systematic review and meta-analysis of randomized clinical trials in adults, adolescents, and children suffering from acute bronchitis, acute rhinosinusitis, or acute tonsillopharyngitis (Matthys et al., 2016) and by a systematic review and meta-analysis of randomized clinical trials in adults with the common cold (Schapowal et al., 2019). Reviews focusing on data from pediatric trials in children and adolescents suffering from different acute respiratory tract infections also found EPs 7630 to be safe in all indications investigated (Careddu and Pettenazzo, 2018; Kamin et al., 2018). Moreover, the good tolerability of *Pelargonium sidoides* was confirmed in a recent network meta-analysis of data from clinical trials in patients suffering from rhinosinusitis (Hoang et al., 2023).

Overall, the available safety analyses show that the risk of adverse events was only slightly increased *versus* placebo level, mainly due to somewhat higher rates of gastrointestinal complaints and hypersensitivity reactions, but the potentially attributable events were always non-serious, non-severe, transient, and not causing an increase in treatment-related withdrawals (Matthys et al., 2013; Keck et al., 2015; Schapowal et al., 2019). Regarding human coronavirus (HCoV) infections, Keck et al. (2021) reported an analysis of data from an open-label trial performed in 2011/2012 and investigating the effects of EPs 7630 in patients with the common cold. Participants tested positive or negative for HCoV showed significant symptom improvements already after 5 days of treatment, with a tendency towards more pronounced improvements in the HCoV-positive patients. For full recovery rates at treatment end (day 10), concomitant antipyretic treatment, and days on sick leave, EPs 7630 treatment outcomes of HCoV-positive patients were comparable to those of HCoV-negative patients. Based on the available clinical and pharmacological data, EPs 7630 may display a promising candidate for the self-management of COVID-19 in addition to self-isolation, rest, hydration, and the use of antipyretics in case of high fever (Silveira et al., 2020; Brendler et al., 2021).

In a scientific brief issued in March 2020, the World Health Organization provided a justification for the off-label use of drugs marketed for other conditions in patients suffering from COVID-19 as long as no approved treatment exists, but also stated that such interventions undertaken by the prescribing physician should be monitored, and that the results should be documented and shared in a timely manner with the wider medical and scientific community (WHO, 2020). However, empirical data in this area are difficult to obtain and sparse.

In the European Union, marketing authorization holders are obliged to monitor the safety of their medicinal products worldwide and record all received reports, i.e., not only those on side effects occurring with intended use but also reports on off-label use. This also applies to off-label use of a medicinal product that does not result in patient’s harm and does not lead to the occurrence of a suspected adverse reaction and includes the documentation of unexpected positive effects of off-label use. One valuable source of information about off-label use may thus be the pharmacovigilance databases of the product manufacturers. Such pharmacovigilance databases contain spontaneous cases, cases originating from published literature, and cases reported in the course of interventional and non-interventional clinical studies. This also applies to off-label use of the manufacturer’s medicinal products that do not result in patient’s harm and occurrence of a suspected adverse reaction.

We therefore performed an analysis of case reports derived from the pharmacovigilance database of the manufacturer of EPs 7630 and indicating the use of products containing EPs 7630 in the context of the treatment or prophylaxis of a COVID-19 infection.

## 2 Methods

We performed a retrospective analysis of cases documented in the Global Pharmacovigilance Safety Database of Dr. Willmar Schwabe GmbH and Co. KG, Karlsruhe, Germany (Schwabe), manufacturer and marketing authorization holder of EPs 7630. The Schwabe Global Pharmacovigilance Safety Database contains spontaneous cases, cases originating from published literature, and cases reported in the course of interventional and non-interventional clinical studies. This also applies to off-label use of EPs 7630 that does not result in patient’s harm and occurrence of a suspected adverse reaction. Reports may be filed by physicians, business partners, patients, or their relatives, as well as health authorities, e.g., via phone, email, letter, or the Schwabe website. According to the European Medicines Agency Guideline on good pharmacovigilance practices (Module VI - Collection, management and submission of reports of suspected adverse reactions to medicinal products), the minimal criteria for valid reports are: one or more identifiable reporter; one single identifiable patient; one or more suspected substance/medical product; and one or more suspected adverse reaction, including special situations such as reports on off-label use with possible beneficial effects (EMA, 2017). Conditions included in reports are coded according to the Medical Dictionary for Regulatory Activities (MedDRA).

An expert at Schwabe Global Pharmacovigilance retrieved the data from the database by means of a computerized search and reviewed the matches. The search covered all cases with mentioning of EPs 7630 (or one of the respected trade names related to EPs 7630 worldwide) reported between 01 December 2019 and 28 February 2023 to identify cases of off-label use of EPs 7630 for the treatment of COVID-19 or post-COVID-19 syndrome. Database queries were based on MedDRA Preferred Terms (PTs) containing one of the following: ‘corona virus infection’, ‘COVID-19’, ‘coronavirus test positive’, ‘SARS-CoV-2 test positive’, ‘SARS-CoV-1 test positive’, ‘coronavirus infection’, ‘COVID-19 prophylaxis’, ‘COVID-19 treatment’, and ‘immune enhancement therapy’. Moreover, a computerized full-text search of the report narratives was carried out for exact matches of the terms ‘coronavirus’, ‘COVID’, ‘COVID-19’, ‘SARS-CoV-2’, ‘SARS CoV 2’, ‘SARS’, and ‘Covid-19’. Included in the analysis were all cases in which EPs 7630 was administered for the treatment or prophylaxis of COVID-19 or post-COVID-19 or for COVID 19-associated complications and concurrent infections, either as monotherapy or in combination with other medicinal products. There were no further exclusion criteria. The database output derived after applying the above-mentioned search specifications (=matches) was then reviewed and classified according to patient age (<18 years, 18–65 years, >65 years, unknown), country of origin, sex, further therapy related to a COVID-19 infection (including concomitant treatments related to other acute respiratory tract infections or their symptoms), indication for EPs 7630 use (COVID-19, Post-COVID syndrome, prophylaxis, other), duration of EPs 7630 treatment, and treatment outcome (improved, worsened, no change, unknown). Moreover, all identified cases were reviewed for adverse events (AEs).

Eligible cases reports were analyzed descriptively for age and sex of the patients, country of origin, indication for EPs 7630 use, duration of EPs 7630 treatment, further medication for COVID-19, and treatment outcome.

## 3 Results

For the period between 01 December 2019 and 28 February 2023, a total of 44 case reports of off-label use of EPs 7630 for COVID-19-related indications were identified in the Schwabe Global Pharmacovigilance Safety Database. With 16 (36.4%) reports, the largest number of cases originated from Germany, followed by 13 (29.5%) from Brazil, 8 (18.2%) from Mexico, and 7 (15.9%) from Italy. A total of 23 (52.3%) reports referred to adults between 18 and 65 years, 2 (4.5%) to patients under the age of 18 years, and 2 (4.5%) to patients over the age of 65 years. In 17 (38.6%) of case reports, the patient age was not indicated. Case reports on female patients (24; 54.5%) were more frequent than on male patients (17; 38.6%). In 3 (6.8%) cases, the respective information was not available.

Overall, 34 out of the 44 reports (77.3%) described cases in which EPs 7630 was administered for the treatment of COVID-19, 7 reports (15.9%) referred to the treatment of post-COVID syndrome, and 3 (6.8%) described prophylactic treatment using EPs 7630. In a total of 15 (34.1%) cases, patients took EPs 7630 for up to 7 days, while 6 (13.6%) were treated for more than 2 weeks. In 22 (50.0%) of the cases, no information about the duration of EPs 7630 treatment was available. Treatment outcome was not reported in 16 (36.4%) cases. All 28 (63.6%) cases reporting on the outcome of EPs 7630 treatment characterized the patients as improved.

Information on whether or not further COVID-19-related treatment was used in addition to EPs 7630 was available in 19 (43.2%) case reports. Further treatment, which was documented in 14 (31.8%) case reports, is depicted in Table 1. For five (11.4%) cases, EPs 7630 monotherapy was reported:

• Two of these patients were tested positive for COVID-19. A favorable outcome was reported for both.
• Two other patients were classified as suffering from COVID-19 based on the reported symptoms. It is not known whether these cases were test-confirmed. One of the patients had a favorable outcome, whereas the outcome of the other is unknown.
• One patient received EPs 7630 as a prophylactic measure against COVID-19.

**TABLE 1:** COVID 19-related further treatment reported in case reports^a^ of off-label use of EPs 7630 for COVID 19-related indications identified in the Schwabe Global Pharmacovigilance Safety Database (coded with ATC; number and proportion of reported further treatment ^b^; December 2019 to February 2023).

Medication	n (%)
Antibiotic NOS	2 (8.3)
Antipyretic NOS	1 (4.2)
Antitussive NOS	1 (4.2)
Amoxicillin/Clavulanate	1 (4.2)
Ascorbic Acid	1 (4.2)
Azithromycin	4 (16.7)
Cetylpyridinium chloride	1 (4.2)
Dexamethasone	1 (4.2)
Expectorants, combinations	1 (4.2)
Ibuprofen	2 (8.3)
Fluticasone furoate	2 (8.3)
Multivitaminic	1 (4.2)
NSAID NOS	3 (12.5)
Steroids NOS	1 (4.2)
Symptomatic treatment NOS	2 (8.3)
**Total**	**24 (100)**

^a^ Further treatment was documented in 14 case reports;

^b^ Patients may have used one or more concomitant medications, which were all counted individually; NOS, not otherwise specified.

Table 2 shows that 30/44 patients (68.2%) did not report any adverse events during treatment with EPs 7630. Complaints reported for more than one patient were gastrointestinal symptoms, hypersensitivity reactions, and headache. Red eyes and blood dyscrasia were each reported once.

**TABLE 2:** Adverse events reported in case reports of off-label use of EPs 7630 for COVID 19-related indications identified in the Schwabe Global Pharmacovigilance Safety Database (preferred terms; number and proportion of reports, n = 44; December 2019 to February 2023).

Adverse event	n (%)
None^a^	30 (68.2)
Gastrointestinal complaints	7 (15.9)
Hypersensitivity reactions	3 (6.8)
Headache	2 (4.5)
Red Eyes	1 (2.3)
Blood dyscrasia	1 (2.3)

^a^ In the European Union, marketing authorization holders are obliged to record all received reports on off-label use of medicinal products. This also applies to off-label use of a medicinal product that does not result in patient’s harm and does not lead to the occurrence of a suspected adverse reaction.

## 4 Discussion

Our review of all cases received in the Schwabe Global Pharmacovigilance Safety Database between the beginning of the COVID-19 pandemic and February 2023 provides a comprehensive summary of all documented information about the off-label use of EPs 7630 for COVID-19-related indications. Even though the total number of documented cases is small, the analysis confirms a certain off-label use of EPs 7630 for COVID-19 in the market.

On the one hand, a systematic review of the extent of underreporting of adverse drug reactions (ADRs) to spontaneous reporting systems has determined a median underreporting rate of 94% across 37 studies, with an interquartile range of 82%–98% (Hazell and Shakir, 2006). Under-reporting is also anticipated to be significant for herbal medications (Chang et al., 2021; Choudhury et al., 2023). Off-label use, on the other hand, has been estimated for prescription drugs to account for 10%–20% of all prescriptions across all indications and patient populations (Fitzgerald and O'Malley, 2014). The rate of underreporting of off-label use to the spontaneous reporting system may thus likely be even substantially higher than for ADRs, particularly when no adverse reactions are observed. This is particularly true for products that are available over the counter, such as those containing EPs 7630, and may therefore be used without seeking medical attention. Thus, the present investigation does not permit an estimate of the extent to which EPs 7630 has been used off-label for COVID-19.

Medicinal products based on *P. sidoides* extracts have been marketed for decades, and their safety profile is well understood (e.g., Matthys et al., 2013; HMPC, 2018). Less than one adverse event report was documented per one million defined daily doses which were brought into the market, resulting in an estimate of one adverse event report per 100,000 treated patients (HMPC, 2018). The available evidence on the safety and tolerability profile of EPs 7630 is based on clinical trials and non-interventional studies including study data from over 10,000 adults and children (Matthys et al., 2013). Case reports available for this analysis do not give rise to any safety concerns in COVID-19 and related indications. While gastrointestinal symptoms and hypersensitivity reactions are known side effects of EPs 7630 according to the Summary of Product Characteristics (SmPC), headache is a common symptom of an acute respiratory tract infection and may thus have been related to the underlying disease. In controlled clinical trials of EPs 7630, headache and abnormal laboratory values were observed with comparable or even lower incidence rates as during treatment with placebo (Matthys et al., 2013). For the event described as ‘blood dyscrasia’, it is not known which laboratory measures may have been involved. However, blood dyscrasias are described as commonly seen in the early stages of viral infections (Demler and Trigoboff, 2009). Finally, ‘red eyes’ is a highly unspecific symptom that may have been attributable to a variety of causes, including an acute respiratory infection.

Regarding the course of COVID-19 during treatment with EPs 7630, it is encouraging that all case reports with a documented treatment outcome, i.e., almost ^2^/_3_ of all available case reports, characterized the patients as improved and that none reported the outcome as unchanged or aggravated. In this context, it is also worth mentioning that treatment outcome was reported for three out of the four patients who received EPs 7630 monotherapy for COVID-19 treatment, and that the outcomes of all three were favorable. A beneficial effect of EPs 7630 on the symptoms associated with COVID-19 would be explainable by the pharmacological profile of the extract, notably by its direct antiviral activity in general (Michaelis et al., 2011; Witte et al., 2015; Roth et al., 2019; Gajewski et al., 2021) and its inhibiting effects on SARS-CoV-2 in particular (Alossaimi et al., 2022; Emanuel et al., 2023; Papies et al., 2021a; b) and may thus not have been unlikely. The observations from the analysis of case reports are also consistent with the results of Keck et al. (2021) who found that EPs 7630 was comparably effective in patients with acute respiratory disorders with or without proven HCoV infection. Even though the HCoV nucleic acid samples in the study of Keck et al. (2021) were isolated prior to the discovery of SARS-CoV-2, the results nevertheless support the interpretation that EPs 7630 could potentially also be effective in COVID-19, a disease caused by a virus from the same virus family.

A shortcoming of our analysis may be that it was based on a very small number of reports that had been collected for an entirely different purpose. Moreover, results have to be considered against the background that there was no control group and that additive effects of other therapies have to be taken into account in cases where EPs 7630 was not taken as a monotherapy. Therefore, our approach has to be regarded as hypothesis-generating, not hypothesis-testing. For the latter purpose, further appropriate research is needed. It would also have been desirable to go deeper into the data and report some aspects not in an aggregated fashion but more explicitly, e.g., describing the duration of use in relation to the reason for taking the medication or presenting adverse events including a more in-depth causality assessment. However, as EU guidelines on data protection are to be considered, it was not possible to go into more detail.

In conclusion, case reports about the off-label use of EPs 7630 that were submitted to the spontaneous reporting system of the manufacturer suggest that the herbal extract may have a beneficial effect in COVID-19-related indications. Evaluating spontaneous reporting of the off-label use of EPs 7630 in COVID-19 is a helpful tool to identify such effects. Given the favorable safety profile of EPs 7630 established over decades of use in acute respiratory tract infections, further assessment of the effects of EPs 7630 in COVID-19 and post-COVID-19 treatment appears to be justified.

## Data Availability

The data analyzed in this study is subject to the following licenses/restrictions: The datasets presented in this article are not readily available because raw data cannot be shared both due to ethical reasons and to data protection laws. All relevant data are within the paper. Requests to access these datasets should be directed to FM, fathi_abdul.malek@schwabe.de.
